# Maxillary sinus floor augmentation and simultaneous dental implant placement in a patient with Guillain‐Barre syndrome: A case report

**DOI:** 10.1002/ccr3.2485

**Published:** 2019-10-17

**Authors:** Fardin Faraji, Mojtaba Bayani, Maryam Jafarpour, Fateme Abdolalian

**Affiliations:** ^1^ Department of Neurology School of Medicine Arak University of Medical Sciences Arak Iran; ^2^ Department of Periodontics School of Medicine Arak University of Medical Sciences Arak Iran; ^3^ Dentist Private Practice Arak Iran

**Keywords:** dental implant, Guillain‐Barre syndrome, maxillary sinus augmentation, osseointegration

## Abstract

Dental implant placement in patients with Guillain‐Barre syndrome could be accomplished, and it may turn into a successful treatment for edentulous sites and functionally stabilized for long life. However, a proper patient selection, accurate medical consultation with physician, atraumatic surgery, and other important cautions should be considered.

## Graphical abstract

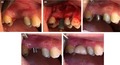

## 1 INTRODUCTION

High success rate has been reported regarding the use of osseointegrated dental implants for the rehabilitation of fully edentulous and partially edentulous jaws as well as replacement of a single missing tooth in the long‐term.^1^ Systemic factors may influence the healing of bone around dental implants. Nevertheless, despite a reduced success rate caused by unfavorable systemic conditions, they may not always be considered as absolute contraindications for bone augmentation and placement of dental implants.^2^ The implant placement and consequently an adequate functional prosthetic rehabilitation in the maxillary molar region require further attention, because of potential bone quality and anatomical structure issues, related to masticatory pattern and occlusal load entity. The reabsorption process, in edentulous posterior maxillary regions, could determine insufficient vertical dimensions for the implant positioning, often requiring additional surgical procedures such as different techniques for maxillary sinus floor augmentation. Several bone graft materials have been used over time for sinus maxillary augmentation.^3^

Guillain‐Barre syndrome (GBS) is an immune‐mediated disorder of the peripheral nervous system, triggered by either infectious or noninfectious factors.^4^ It is the most common form of acute flaccid paralysis occurring at any age. The incidence of GBS increases with age after 50‐year old from 1.7/10 000/y to 3.3/100 000/y.^5^ Controlled epidemiological studies have reported that GBS is associated with infection with Campylobacter jejuni in addition to viruses, including cytomegalovirus and Epstein‐Barr virus.^6^ This lethal syndrome is characterized by muscle weakness and paralysis starting in the lower extremities and progress in an ascending fashion to the gastrointestinal and respiratory muscles and upper limbs.^7^ There are no strong clinical trials with respect to dental implant placement guidelines in patients with GBS, and this treatment is not absolutely contraindicated in them. But some problems in dental implant treatment of patients with GBS, as well as the major precautions are noticed below:

- Autonomic symptoms occur in about two‐thirds of patients with GBS, and they include cardiac arrhythmias, orthostatic, and blood pressure instability.^8^ The sequential treatment plan generally starts with consulting the physician regarding the current medical status, medication, and patient management during dental implant surgery. Dentists must inform the physician regarding the estimated degree of stress, length of procedures, and complexity of the individualized treatment plan.
- The etiology of GBS might be multifactorial and any dentistry procedures such as dental implant surgery may stimulate risk of GBS recurrence. Sporadic cases of GBS have been reported after maxillofacial surgery.^9^ Therefore, a meticulous consultation with patient's physician before any surgical procedures is necessary and in order to decrease traumatic injury during dental implant surgery, the minimally invasive surgery protocols must be implemented.
- GBS is characterized by progressive weakness which rapidly ascends to the muscles of upper body and face,^10^ and it may involve some important masticatory muscles such as buccinators or masseter.^11^ So replacement of missing teeth in GBS patients by dental implants can improve the function of mastication in patients with GBS. However, any replacement of missing teeth by removable prosthesis is not recommended (as it may cause poor neuromuscular coordination during chewing, with altered muscular movement patterns); it is logical to consider fixed prosthesis treatment for rehabilitation of fully or partially edentulous area.
- Up to 80% of patients with GBS experience persistent and severe fatigue after resolution of other GBS symptoms.12 It is an important matter to be noticed during dental implant treatment, dentist should allow patients to attain a comfortable position in a dental chair, initiate breaks (involving closing their mouth and resting), and set dental appointments in short time.

In this regard, a successful case of sinus floor augmentation and simultaneous dental implant placement in a patient with GBS is considered as one of the rare successful cases in oral implantology field.

## 2 CASE PRESENTATION

A 28‐year‐old woman referred to the private dental clinic with complaint about difficulty in mastication. She was a nonsmoker and was diagnosed with GBS 24 months ago. She declared former earlier signs and symptoms such as progressive fatigue, hypoactivity, slurred speech, multiple episodes of choking, urinary incontinence, difficulty breathing, drooling of saliva, and ascending weakness that led to inability to bear her weight so was observed. No reduced tendon reflexes were observed. All blood examination parameters including Na and K were normal, and no abnormality was seen in brain and spinal cords MRI. In EMG‐NCV of four organs, acute signs of motor‐sensory polyneuropathy were observed.

Patient was admitted to ICU and treated by IVIG 30 gr/daily, for 5 days. Vital signs of patient were under control. The patient's symptoms slowly subsided after receiving medications and intensive cares was discharged and was under supportive care by occupational and physiotherapy. In later examinations, the patient was able to walk, eat, and swallow and had no respiratory or autonomic disorders but had a little debility in distal motion of her ankle.

Pre‐operative examination of her oral mucosa revealed no evidence of pathological lesions, and overall oral hygiene was found to be good. The patient felt healthy at the time of examination, and she was well‐nourished, alert, and cooperative. Every surgical and prosthetics procedure was performed after medical consultation with her neurologist.

After meticulous consulting sessions with the patient and discussing the advantages and disadvantages of dental implant treatment, she accepted to receive this treatment and written informed consent was obtained from her.

Before surgery, the patient was premeditated with 2 g of amoxicillin/clavulanic acid and 50 mg of diclofenac. The bone graft was placed into the sinus cavity following a lateral maxillary sinus wall technique. The first stage involved placing the bone graft materials [Demineralized Freeze‐Dried Bone Allograft (DFDBA, Tissue Regeneration Corporation) and a collagen membrane (with 0.6‐0.9 of thickness Hamanand Saz Baft)] before the fixture insertion, so as to be able to reach the medial wall and thus easily compact the bone graft materials. The remaining graft was placed after situating the root form titanium dental implant (Dio pars, Dio, South Korea) with 5‐mm diameter and 10‐mm length in their final position. The patient well tolerated the surgical procedure and her vital signs were regularly monitored. Postoperative medications including antibiotics (1000 mg of amoxicillin/clavulanic acid twice daily for seven days, starting on the day of surgery), an analgesic (600 mg of ibuprofen as required every 6 hours), and mouthwash (0.2% chlorhexidine twice daily for 2 weeks, starting on the day after surgery) were prescribed to the patient. No remarkable complication was observed during postoperative course, and healing and everything were found to be typical. She was instructed to resume normal oral hygiene and chewing by 6th week. Postsurgical cleaning protocols, including oral hygiene instructions, were implemented at weeks 1, 2, 6, and 12.

**Figure 1:**
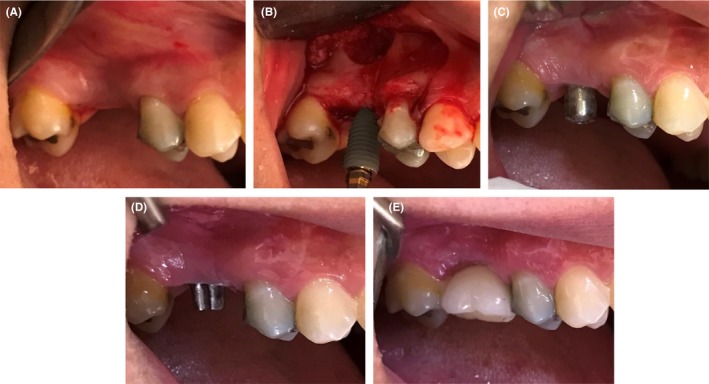
Clinical procedure of dental implant placement: A, implant site in baseline; B, open sinus floor augmentation and fixture placement; C, healing abutment insertion after 6 mo; D, final prosthetic abutment placement, and E, final restoration

**Figure 2:**
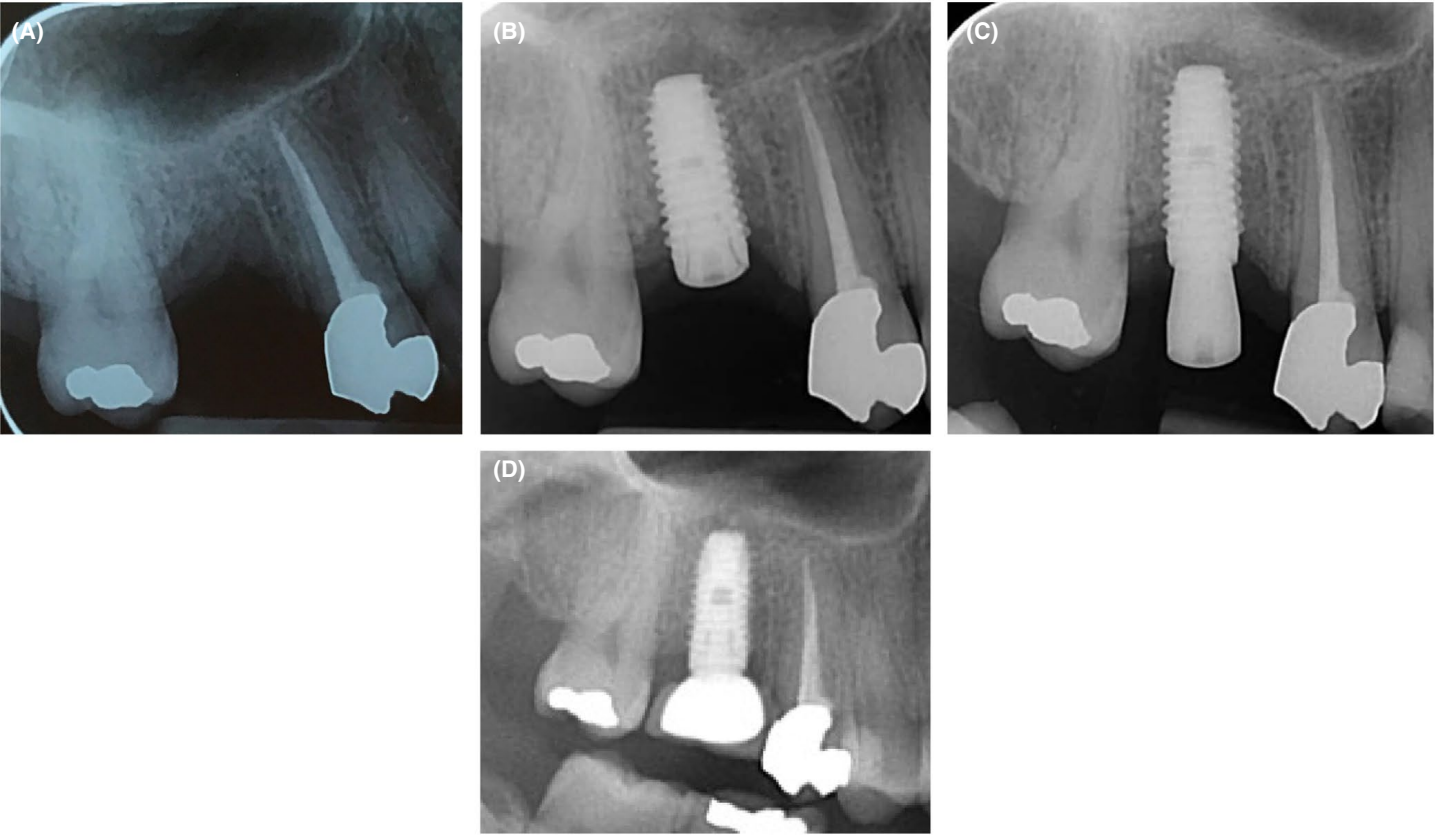
Intraoral radiographs of dental implant placement: A, baseline; B, on the day of implant insertion; C, after 6 mo, and D, last follow‐up after 20 mo

Six months after the implant insertion, the patient returned for punch removal of the gingiva overlying the implant. The patient was anesthetized under local anesthesia following the re‐entry procedure, and healing abutment was placed on the implant for appropriate gingival formation around the implant. After 2 weeks, the appropriate impression coping was connected to the fixture. Polyether (Permadyne light and regular body; ESPE) was injected around the transfer coping and was placed inside the custom tray using the dispenser. After laboratory procedure, abutment was positioned and torqued according to the manufacture's guidelines at 35 Ncm. The patient was rehabilitated by a cemented porcelain fused metal prosthesis, enabling retrievability by the dentist. The occlusal contacts were evenly distributed over the arch through group function contact protocol. After the surgical and prosthetic treatments were completed in September 2017 (Figures 1 and 2), the patient was placed on a regular follow‐up for peri‐implant maintenance. The mechanical oral hygiene regimen was implemented for this patient in a 6‐month recall. The last follow‐up (20 months after prosthetic delivery) showed minimum bone loss, compared with the X‐rays taken immediately after the prosthetic delivery; the implant and its restoration were found to be successful. The patient was satisfied with the treatment in all regular follow‐up sessions.

## 3 DISCUSSION

The tooth replacement by dental implant has become a more attractive and efficient alternative, compared with the conventional fixed and/or removable dental prosthetics.^12^ Some systemic conditions influence the quality or quantity of jawbones, osseointegration events, and optimal soft or hard tissue healing sequences after implant placement. They are also considered to be related or absolutely contraindications for implant treatment.^1^, ^2^

Three significant problems were found in this study, which could hinder the insertion of dental implant in safe condition: (a) GBS might be influenced by bone quality and biology in the patient; (b) GBS or its relative medication can be influenced by immunological events and this patient was very susceptible to alter in inflammatory response in osseointegration period leading to early implant failure; (c) need to maxillary sinus floor augmentation for improving the vertical bone height before implant insertion in the present case, and we performed this treatment into a complex surgical procedure instead of a simple tooth implantation and increased potential failure risks of dental implant.

We could use close sinus lift procedure with osteotome technique for the improvement of vertical dimension in implant site, but a major concern for using osteotome technique in GBS patients is the probability of trauma due to stroke of osteotome and increased risk of GBS.^13^ The present case study was conducted based on the following criteria: the permission of the patient's physicians after multiple medical consultation; proper bone quality assessed by pre‐operative imaging; proper tactile sense of surgeon during implant site preparation; achievement of good primary stability; lack of any soft and hard tissue lesions in the surgical site or any periodontal pathology; not having or no history of any other systematic disease altering osseointegration events such as diabetes; good oral hygiene; implementation of conservative implant surgery protocols; maxillary sinus augmentation in small sites and minimized aggression to sinus environment; performing all surgical and prosthetically sessions with minimally chair side time and placing the patient in proper dental unit position between each implant procedures; lack of any obvious mechanical impairment in patient before implant treatment; adequate patient cooperation; and lack of strong evidence stating that dental implant in patients with GBS is absolutely contraindicated.

The implant therapy has been followed for 20 months, and the patient is currently healthy. Minimum amount of bone loss was observed around the implant. The results of the study revealed that conservative and careful selection of patients with GBS along with thorough proper medical consultation with the patient's physician in addition to optimal timing of the surgical and restoration phases of the implant treatment led to a successful outcome regarding the dental implant treatment. To the best of our knowledge, this is the first report describing sinus floor augmentation and simultaneous dental implant insertion in a patient with GBS.

## 4 CONCLUSION

Although dental implant therapy showed an excellent result in the present case, it would be of great benefit to determine the predictability of conservative implant treatments in patients with GBS. Long‐term clinical trials on the placement of implants in patients with GBS are required to establish clear clinical guidelines. These guidelines would be based on well‐conducted clinical trial studies with exact and proper study designs for the management of adverse side effects caused by GBS process and alteration in wound‐healing events due to medications. Dental implant can be osseointegrated and remain functionally stable in patients with GBS.

## CONFLICT OF INTEREST

The authors declare that they have no conflict of interests.

## AUTHOR CONTRIBUTIONS

MB: performed dental implant insertion and its prosthodontics procedure, and designed and implemented this case. FF: developed theoretical framework, monitored patient's health status, and supervised this report. MJ and F A: contributed in writing and editing of this manuscript.

## ETHICS APPROVAL AND CONSENT TO PARTICIPATE

This case report was approved by the Ethical Practices Committee of Arak University of Medical Sciences, with reference number: IR. ARAKMU.REC.1396.024, and this patient gave informed consent.

## CONSENT FOR PUBLICATION

Written informed consent was obtained from the patient for the publication of this case report and any accompanying images. A copy of the written consent is available for review by the Editor‐in‐Chief of this journal as an additional file.

## Data Availability

The datasets used and/or analyzed during the current report are available from the corresponding author [Dr Mojtaba Bayani] on reasonable request.
